# Side population cells have the characteristics of cancer stem-like cells/cancer-initiating cells in bone sarcomas

**DOI:** 10.1038/sj.bjc.6605330

**Published:** 2009-09-29

**Authors:** M Murase, M Kano, T Tsukahara, A Takahashi, T Torigoe, S Kawaguchi, S Kimura, T Wada, Y Uchihashi, T Kondo, T Yamashita, N Sato

**Affiliations:** 1 Department of Orthopaedic Surgery, Sapporo Medical University School of Medicine, South-1, West-16, Chuo-ku, Sapporo 060-8543, Japan; 2 Department of Pathology, Sapporo Medical University School of Medicine, South-1, West-17, Chuo-ku, Sapporo 060-8556, Japan; 3 Cancer Vaccine Laboratory, Japan Science and Technology Corporation, North-19, West-11, Kita-ku, Sapporo 060-0819, Japan; 4 Team for Cell Linage Modulation, RIKEN Center for Developmental Biology, 2-2-3, Minatojima-minamimachi, Chuo-ku, Kobe 650-0047, Japan

**Keywords:** cancer stem-like cell, cancer-initiating cell, osteosarcoma, bone malignant fibrous histiocytoma, side population

## Abstract

**Background:**

Several human cancers have been found to contain cancer stem-like cells (CSCs) having cancer-initiating ability. However, only a few reports have shown the existence of CSCs in bone and soft tissue sarcomas. In this study, we identified and characterised side population (SP) cells that showed drug-resistant features in human bone sarcoma cell lines.

**Methods:**

In seven osteosarcoma cell lines (OS2000, KIKU, NY, Huo9, HOS, U2OS and Saos2) and in one bone malignant fibrous histiocytoma (MFH) cell line (MFH2003), the frequency of SP cells was analysed. Tumourigenicity of SP cells was assessed *in vitro* and *in vivo*. Gene profiles of SP cells and other populations (main population; MP) of cells were characterised using cDNA microarrays.

**Results:**

SP cells were found in NY (0.31%) and MFH2003 (5.28%). SP cells of MFH2003 formed spherical colonies and re-populated into SP and MP cells. In an NOD/SCID mice xenograft model, 1 × 10^3^ sorted SP cell-induced tumourigenesis. cDNA microarray analysis showed that 23 genes were upregulated in SP cells.

**Conclusions:**

We showed that SP cells existed in bone sarcoma cell lines. SP cells of MFH2003 had cancer-initiating ability *in vitro* and *in vivo*. The gene profiles of SP cells could serve as candidate markers for CSCs in bone sarcomas.

Over the past three decades, there have been remarkable advances in the treatment of bone and soft tissue sarcomas. These include the introduction of adjuvant chemotherapy, establishment of guidelines for adequate surgical margins and the development of post-excision reconstruction (Fletcher *et al*, 2002; Lewis, 2007). However, the prognosis of non-responders to chemotherapy is still poor and the mechanisms of tumourigenesis of bone and soft tissue sarcomas remain to be demonstrated.

Generally, cancer masses are considered to be a complex of heterogeneous but equally malignant cell populations. However, recent stem cell research on the development of normal organs has drawn attention to the existence of a ‘cancer stem-like cell (CSC)’ counterpart, which is characterised by its self-renewal capacity, differentiation potential, and cancer-initiating ability (Visvader and Lindeman, 2008). On the basis of these characteristics, CSCs have been postulated to be responsible for driving the growth of tumours and for the recurrence of neoplasms after current therapeutic modalities are used.

Initial attempts to characterise CSCs were accomplished using cell surface molecules in acute myeloid leukaemia. Several groups that found CSCs capable of initiating leukaemia were found in the CD34^+^CD38^−^ fraction (Warner *et al*, 2004). Recently, CSCs have been isolated from several human solid tumours that have markers for putative normal stem cells, including breast cancer (CD44^+^CD24^−^ESA^+^) (Al-Hajj *et al*, 2003), pancreatic cancer (CD44^+^CD24^−^ESA^+^, CD133^+^CXCR4^+^) (Hermann *et al*, 2007; Li *et al*, 2007), brain cancer (CD133^+^) (Singh *et al*, 2004), prostate cancer (CD44^+^/*α*_2_*β*_1_^hi^/CD133^+^) (Collins *et al*, 2005), hepatocellular carcinoma (CD133^+^) (Yin *et al*, 2007) and colon cancer (CD133^+^) (Ricci-Vitiani *et al*, 2007).

On the other hand, in the analysis of haematopoietic stem cells, a sub-population that effluxes the DNA-binding dye Hoechst 33342 out of the cell membrane through an ATP-binding cassette (ABC) transporter was recognised as a stem cell population (Goodell *et al*, 1996; Zhou *et al*, 2002; Robinson *et al*, 2005). This cell population expressing the ABC transporter was defined as side population (SP) cells, which were distinguished from cells of the other population (main population; MP). Recent studies demonstrated that SP cells could be characterised as CSCs in primary tissues of gastrointestinal cancers (Haraguchi *et al*, 2006) and ovarian cancer (Szotek *et al*, 2006). SP cells were also shown in established tumour cell lines with different origins, such as glioma (Kondo *et al*, 2004), breast (Kruger *et al*, 2006) and thyroid cancer monoclonal cell lines (Mitsutake *et al*, 2007).

To date, however, distinct molecular markers on CSCs are still lacking in many cancers. Moreover, only a few reports have shown the existence of CSCs in bone and soft tissue sarcomas (Gibbs *et al*, 2005; Wu *et al*, 2007; Tirino *et al*, 2008). In this study, with the goal of determining specific markers of CSCs, we identified and characterised SP cells having cancer-initiating ability in osteosarcoma and bone malignant fibrous histiocytoma cell lines.

## Materials and methods

### Cell lines and culture

Seven human osteosarcoma (OS) cell lines (OS2000, KIKU, NY, Huo9, HOS, U-2OS and Saos2) and one bone human malignant fibrous histiocytoma (MFH) cell line (MFH2003) were used. OS2000, KIKU and MFH2003 were established in our laboratory (Wada *et al*, 1988; Nabeta *et al*, 2003; Tsukahara *et al*, 2006). The other cell lines were kindly donated by or purchased from the Japanese Collection of Research Bioresources Cell Bank (Tokyo, Japan) and from the American Type Culture Collection (Manassas, VA, USA). MFH2003 and OS2000 were cultured with Iscove's modified Dulbecco's Eagle's medium (IMDM; GIBCO BRL, Grand Island, NY, USA) containing 10% FBS and the others were maintained in Dulbecco's modified Eagle's medium (DMEM; Sigma-Aldrich, St Louis, MO, USA) containing 10% FBS in a 5% CO_2_ incubator at 37°C.

### Identification of side population

Cell suspensions were labelled with dye Hoechst 33342 (Cambrex Bio Science Walkersville Inc., MD, USA) using the methods described by Goodell *et al* (1996) with some modifications . Briefly, cells were trypsinised and re-suspended in pre-warmed DMEM supplemented with 5% FBS at a concentration of 1 × 10^6^ ml^−1^. Hoechst33342 dye was added at a final concentration of 2.5 or 5.0 *μ*g ml^−1^ in the presence or absence of verapamil (50 or 75 *μ*M; Sigma-Aldrich) as an inhibitor of the ABC transporter. The cells were incubated at 37°C for 90 min with continuous shaking. At the end of the incubation, the cells were washed with ice-cold PBS with 5% FBS, centrifuged at 4°C and resuspended in ice-cold PBS containing 5% FBS. Propidium iodide (at a final concentration of 1 *μ*g ml^−1^; Molecular Probes–Invitrogen, Eugene, OR, USA) was used to gate viable cells. Flow cytometry and cell sorting were performed using FACSVantage SE (BD Biosciences, Bedford, MA, USA), EPICS ALTRA (Beckman-Coulter, Fullerton, CA, USA) and FACS Aria II (BD Biosciences). The Hoechst 33342 dye was excited at 357 nm and its fluorescence was analysed using dual wavelengths (blue, 402–446 nm; red, 650–670 nm).

### RNA preparation

Total RNAs were extracted from SP cells and MP cells using the RNeasy Mini kit (Qiagen, Hilden, Germany) according to the manufacturer's protocol. Using an Amino Allyl MessageAmp aRNA Kit Ver. 2 (Sigma-Aldrich Japan, Ishikari, Japan), amino allyl-modified aRNAs were prepared from total RNAs from SP and MP cells as previously described (Takeuchi *et al*, 2008).

### Real-time PCR analysis

Total RNA was reverse transcribed using the SuperScriptIII reverse transcriptase enzyme (Invitrogen) according to the manufacturer's instructions. Real-time PCR was performed with SYBR Green Real-time Core Reagent (Applied Biosystems, Foster City, CA, USA) according to the manufacturer's instructions on an ABI Prism 7900 Sequence Detection System (Applied Biosystems). Primers were designed to generate a PCR product of <200 bp. The thermal cycling conditions were 94°C for 2 min, followed by 35 cycles of 15 s at 94°C, 30 s at 60°C and 30 s at 72°C. Levels of expression were normalised to the *glyceraldehyde-3-phosphate dehydrogenase* (G3PDH) housekeeping gene.

### Spherical colony formation assay

Spherical colony formation assay was performed as described by Gibbs *et al* (2005) with some modifications. Briefly, cells were plated at 2000 cells per well in six-well ultra-low attachment plates (Corning Inc., Corning, NY, USA). Mesenchymal Stem Cell Basal Medium (MSCBM) and MSCBM SingleQuots (Takara Bio Inc., Ohtsu, Japan were used for cell culture. Fresh aliquots of epidermal growth factor and basic fibroblast growth factor were added every other day. On day 14, the numbers of colonies were counted.

### Xenograft model

Sorted SP and MP cells of MFH2003 were collected and re-suspended at 1 × 10^2^–1 × 10^5^ cells per 50 *μ*l of PBS, followed by addition of 50 *μ*l of Matrigel (BD Biosciences). This cell-Matrigel suspension was subcutaneously injected into the backs of 4- to 6-week-old non-obese diabetic/severe combined immunodeficiency (NOD/SCID) mice (NOD.CB17-Prdkc^scid^/J, Charles River Laboratory, Yokohama, Japan) under anaesthesia. Mice were observed for up to 12 weeks.

### Gene expression profiling using cDNA microarrays

The aRNAs from SP cells were labelled with Cy5 dye and those from MP cells were labelled with Cy3 dye. The dye-labelled aRNA samples were hybridised to a 29 138-spot Human Panorama Micro Array (Sigma-Aldrich) for 16 h at 45°C. The intensities of Cy5 and Cy3 fluorescence for every gene spot on the hybridised array were measured with a GenePix 4000B scanner (Axon Instrument, Austin, TX, USA), and were analysed with GenePix Pro 5.0 software (Axon Instrument). Global normalisation of the resultant data was carried out using Excel 2004 (Microsoft, Redmond, WA, USA). As a result, 24 730 genes were available for further analysis. The average expression ratio of Cy5 to Cy3 was obtained for each gene. A dye-swap experiment (labelling SP and MP cells with Cy3 and Cy5, respectively) was also performed. An average ratio of more than 2.0, reproducible in two experiments, was determined to indicate differential upregulation in SP cells.

## Results

### Identification of SP cells in human bone sarcoma cell lines

**Figure 1:**
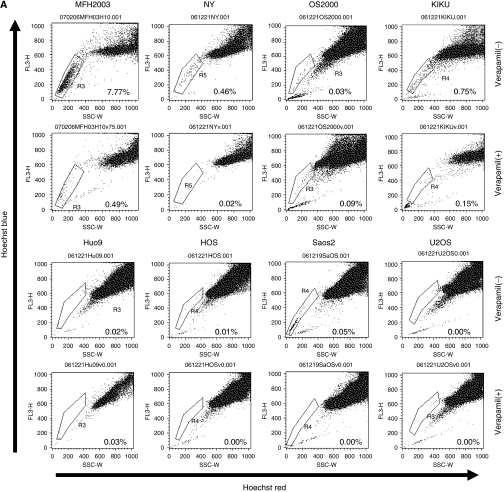

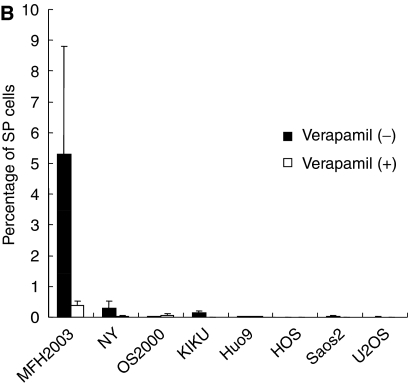
Detection of side population cells in bone sarcoma cell lines. (**A**) The populations of SP cells of seven osteosarcoma cell lines (NY, OS2000, KIKU, Huo9, HOS, Saos2 and U2OS) and of one bone MFH cell line (MFH2003), in the presence or absence of verapamil, are shown. SP cells are marked by black dotted lines to show the proportion of SP cells among total living cells. (**B**) The mean proportions of SP cells in cell lines. These results were reproducible in at least two independent experiments.

To identify the CSCs of bone sarcomas, we tried to detect side population (SP) cells in bone and soft tissue sarcoma cell lines. As depicted in Figure 1A, the NY and MFH2003 cell lines included 0.38 and 5.28% SP cells, respectively. In each case, the percentage of SP cells was markedly diminished by treatment with verapamil, which is an inhibitor of the pumps responsible for the exclusion of Hoechst dye, indicating that this population truly represented SP cells. SP cells were hardly detected in the other cell lines (Figure 1B). Therefore, MFH2003, containing the highest proportion of SP cells, was selected and further analysed.

### Isolation of SP cells and their repopulation as both SP and MP cells

**Figure 2:**
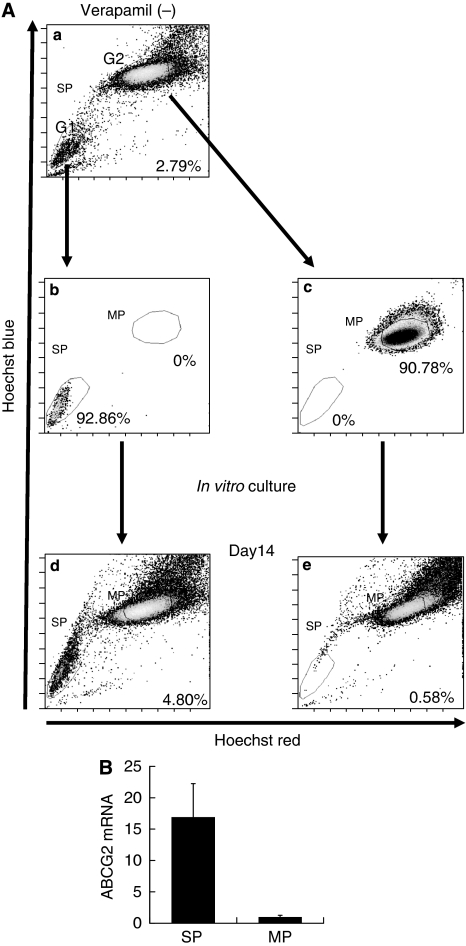
The re-population of SP cells into both SP and MP cells. (**A**) (**a**) The populations of SP cells and MP cells before cell sorting are shown. SP cells were gated as G1, and MP cells were gated as G2. (**b**, **c**) The proportions of SP cells among the total living cells are indicated. Isolated SP cells (**b**) and MP cells (**c**) after cell sorting. The proportions of SP and MP cells among the total living cells are indicated. (**d**, **e**) The populations of SP cells (**d**) and MP cells (**e**) after 2-week *in vitro* culture with medium containing 10% FBS are also shown. Experiments were repeated in triplicate with similar results. (**B**) The relative expression of ABCG2 was evaluated in SP cells and MP cells by real-time PCR.

After excluding dead cells and cellular debris on the basis of scatter signals and propidium iodide fluorescence, MFH2003 cells were sorted into SP and MP cells. As shown in Figure 2A, the G1 gate showed the SP cells that had negative/low patterns of staining with Hoechst 33342, and the G2 gate showed the main population cells that were positively stained with Hoechst 33342. To ascertain the purity of sorted cells, the obtained SP (G1) cells and MP (G2) cells were re-analysed. The purity levels were 92.86 and 90.78% in the SP population and MP population, respectively. These results supported the specificity for further analysis using the resultant SP and MP cells.

To examine whether SP cells could generate both SP and MP cells, sorted SP and MP cells were further cultured *in vitro*. On day 14, the cells were re-stained with Hoechst 33342 and analysed by flow cytometry. We observed that SP cells re-populated both SP and MP cells. The ratio of SP cells to MP cells was still much higher than that before sorting. In contrast, SP cells were not detected in sorted MP cells.

Expression of *ABCG2* mRNA, which is a marker of SP cells, was increased in SP cells (Figure 2B). These results also supported the specificity for further characterisation of SP cells, especially with regard to their cancer-initiating ability.

### Spherical colony formation

**Figure 3:**
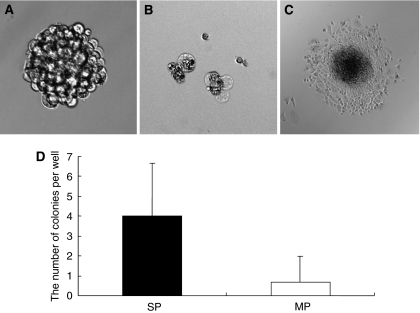
Tumourigenesis of SP and MP cells *in vitro*. (**A** and **B**) The features of spherical colonies derived from resultant SP cells (**A**) and MP cells (**B**) cultured without serum in an anchorage-independent manner for 2 weeks. (**C**) Spherical colony removed from the suspension culture and allowed to attach to a substratum. Adherent cells can be seen expanding from the sphere. (**D**) The numbers of resultant spherical colonies from SP cells and MP cells were counted. Data are representative of three independent experiments.

We next evaluated the ability of SP and MP cells to generate spherical colonies. A total of 2000 SP and MP cells were sorted and cultured immediately under conditions of serum starvation, providing an anchorage-independent environment. On day 14, SP cells showed spherical colony formation (Figure 3A). On the other hand, as shown in Figure 3B, most MP cells died and the others formed a few small colonies. We removed spherical colonies from the suspension culture and attempted again to determine whether the cells could attach to a substratum. As shown in Figure 3C, cells were seen expanding from the sphere. In Figure 3D, the number of colonies is shown, indicating clearly that, among MFH2003 cells, SP, but not MP, cells had the potential for spherical colony formation.

### Cancer-initiating ability of SP and MP cells *in vivo*

**Figure 4:**
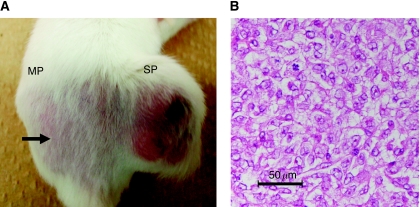
The features of xenotransplanted SP cells *in vivo*. (**A**) Macroscopic features of 1 × 10^3^ each of SP and MP cells in an NOD/SCID mouse at 12 weeks after xenotransplantation. Black arrow indicates the site of injection of MP cells. (**B**) Histological findings of the xenotransplanted tumour derived from SP cells (1 × 10^4^). Haematoxilin and eosin staining (original magnification: × 200) is shown.

To address the issue of whether tumourigenic activity differed between SP and MP cells, 1 × 10^2^–1 × 10^5^ SP and MP cells sorted from MFH2003 were injected into NOD/SCID mice (Figure 4A). To rule out the effects of the toxicity of Hoechst, we routinely performed (i) depletion of dead cells by PI staining and (ii) a viability check using trypan-blue staining after cell sorting. Almost all MP cells were viable as SP cells. Subcutaneous tumour formation was induced by the injection of 1 × 10^3^ SP cells (Table 1). We also observed that 1 × 10^4^ SP cells formed a larger tumour mass than did 1 × 10^3^ SP cells (data not shown). In contrast, at least 1 × 10^5^ MP cells were required to give rise to a tumour. Macroscopic and microscopic findings of a tumour derived from SP cells are shown in Figure 4A and B. These results supported the hypothesis that SP cells have a high cancer-initiating ability, similar to CSCs. At 8 weeks after xenotransplantation, the frequencies of SP and MP cells in a formed tumour derived from 1 × 10^4^ SP cells were analysed *ex vivo*. SP cells were hardly detected in the tumour. Most SP cells re-populated into MP cells *in vivo* in 8 weeks (data not shown).

### Identification of upregulated genes in SP cells

Finally, we performed gene expression profiling of SP cells using cDNA microarrays. As shown in Table 2, 23 genes were found to be upregulated in SP cells, compared with MP cells. Although the functions of upregulated genes varied, these results suggested that the factors connected to DNA transcription, transport of substrates, cell proliferation and apoptosis might have a role in the cancer-initiating ability of SP cells. In addition, the increased expression of *ABCG2* in SP cells confirmed the accuracy of the gene expression profiling analysis.

## Discussion

In this study, we showed that (i) small SP populations existed in one osteosarcoma cell line and one bone MFH cell line; (ii) SP cells derived from MFH2003 could re-populate both SP and MP cells *in vitro*; (iii) SP cells could form spherical colonies and re-populate into SP and MP cells; (iv) SP cells had tumourigenesis in an *in vivo* xenograft model; and (v) factors regarding transcription, cell proliferation and apoptosis were upregulated in SP cells.

We observed proportions of SP cells of 0.31 and 5.28% in NY and MFH2003, respectively. The proportions of SP cells we observed were similar to those in most previous reports, with 2% noted in human breast cancer cell line MCF7, 0.4% in rat C6 glioma, 1.2% in human HeLa carcinoma (Kondo *et al*, 2004) and 4–37% in neuroblastoma cell lines (Hirschmann-Jax *et al*, 2004).

The SP cells were defined by the efflux of Hoechst 33342, a cell-permeable DNA-specific bisbenzimidazole dye, through an ABC transporter. Therefore, SP cells are considered to be resistant to multi-chemotherapeutic drugs and to confer malignant phenotypes to tumours (Dean *et al*, 2005). Hence, the characterisation of SP cells might be a useful tool for analysis of CSCs, especially when specific CSC surface markers are unknown.

We found that SP cells could re-populate both SP cells and MP cells *in vitro*. These results suggested that SP cells were capable of self-renewal and also generated MP cells by asymmetric division. This indicated that a tumour hierarchy might exist in bone MFH. Previous studies have also shown that SP cells can divide asymmetrically and display a capacity for self-renewal similar to normal stem cells (Kondo *et al*, 2004; Singh *et al*, 2004). On the other hand, we also observed that most SP cells xenotransplanted in NOD/SCID mice re-populated into MP cells *in vivo*. SP cells might hardly be maintained *in vivo* for long time, that is, more than 8 weeks. In other words, the niche of the mouse model might not be adequate for the maintenance of SP cells derived from bone sarcoma cell lines, such as MFH2003.

The ability of SP cells to generate spherical colonies was higher than that of MP cells. This is consistent with previous studies (Patrawala *et al*, 2005; Mitsutake *et al*, 2007). We recognised that the difference was not a consequence of longer retention of Hoechst dye in MP cells, because MP cells were viable after staining with the dye, followed by sorting and maintenance in a culture medium with FBS. However, we cannot completely rule out the possibility that the difference was due to some effect of the Hoechst dye, which is potentially cytotoxic (Durand and Olive, 1982).

We could detect a higher tumourigenic potential of SP cells than of MP cells *in vivo* using a NOD/SCID xenograft model. In the field of bone and soft tissue sarcoma, only Wu *et al* (2007) succeeded in showing the *in vivo* cancer-initiating ability of CSCs derived from bone and soft tissue sarcomas, using SP cells isolated from fresh primary tumour tissues. The ability to consistently isolate MFH2003-derived SP cells allowed us to conduct SP cell-specific gene profiling. Moreover, it might become possible to identify CTL-defined CSC-specific tumour antigens for immunotherapy targeting CSCs. Thus far, we have been trying to identify CTL-defined tumour antigens by forward and reverse immunological approaches and have carried out antigenic peptide vaccination trials in bone and soft tissue sarcomas (Sato *et al*, 2002; Ida *et al*, 2004; Tsukahara *et al*, 2004, 2008a, 2008b, 2009; Kawaguchi *et al*, 2005; Kimura *et al*, 2008). Currently, we are trying to establish autologous CTL clones recognising SP cells of MFH2003 from tumour-infiltrating lymphocytes.

Thus far, only Oct3/4, Nanog and CD133 were reported to be candidates for CSC-specific markers in bone and soft tissue sarcoma (Gibbs *et al*, 2005; Tirino *et al*, 2008). Therefore, the gene profile of SP cells might help to expand the possibility of an effective isolation of CSCs from bone and soft tissue sarcomas using these specific markers. In the current gene expression profiling, 23 genes with various functions were upregulated in SP cells. Among them, eight genes (*VPF*, *c20orf14*, *MCL1*, *NR4A2*, *IRX3*, *NRLP12*, *PTN* and *LMNA*) might be considered to be potential tumourigenic factors in malignancies. VPF, generally known as vascular endothelial growth factor, regulates vascular permeability, angiogenesis, cell migration and apoptosis in tumours (Nagy *et al*, 2008). C20orf14 is upregulated in lymphoma (Su *et al*, 2008) and HPV16/18-positive cervical cancer (Vazquez-Ortiz *et al*, 2007). MCL1 is a member of the B-cell lymphoma (BCL) family. MCL-1 negatively regulates pro-apoptotic factors (Bak and Bax) (Chen *et al*, 2007) and accelerates leukaemogenesis (Beverly and Varmus, 2009). NR4A2 belongs to the steroid nuclear hormone receptor superfamily and has a role in cell transformation in cervical cancer (Ke *et al*, 2004). IRX3 is epigenetically inactivated by methylation in CpG islands in brain tumours (Ordway *et al*, 2006) and prostate cancer (Morey *et al*, 2006). NLRP12, also known as RNO2/monarch-1, is reported to activate inflammation in humans (Ye *et al*, 2008). PTN is an angiogenic factor that stimulates tumour-associated vascular formation in many malignancies. (Perez-Pinera *et al*, 2008). LMNA is reported to encode lamin A, which is a putative colonic epithelial stem cell marker and is also a prognostic factor in colorectal cancer (Willis *et al*, 2008). On the other hand, four genes (*ANKRD11*, *PHLDA3*, *APOL1* and *MSX1*) are known as tumour-suppressor factors. ANKRD11 is a p53-interacting protein and activates the transcription of p53 in breast cancer. PHLDA3 is a positive regulator of Fas-dependent death signalling, related to cisplatin-mediated apoptosis (Kerley-Hamilton *et al*, 2005). APOL1 is classically thought to be involved in lipid transport and metabolism and has rarely been characterised with regard to cell survival. Although the structure of APOL1 is similar to that of the anti-apoptotic proteins of the Bcl-2 family (Vanhollebeke and Pays, 2006), it can induce autophagic cell death (Wan *et al*, 2008). MSX1, a homeobox gene important for embryonic neural crest development, can induce the inhibition of tumour-initiating ability in soft agar *in vitro*. Taken together, the gene expression profiling in SP cells derived from MFH2003 containing various tumour-proliferative and tumour-suppressive factors might indicate the complexity of maintaining the characteristics of SP cells as tumour initiators. However, considering the characteristics of SP cells for cancer-initiating ability, the apoptosis-related molecules among these genes (MCL-1, ANKRD11, PHLDA3 and APOL1) might have roles in the proliferation of SP cells. Moreover, these molecules could be candidates for specific markers and, in addition, molecular therapeutic targets.

In conclusion, we identified SP cells in established human bone sarcoma cell lines. Moreover, we demonstrated that bone MFH-derived SP cells can re-populate both SP and MP cells and have cancer-initiating ability *in vitro* and *in vivo*. These findings supported the idea that bone sarcomas might contain a certain population of CSCs. Gene profiling of SP cells could serve to elucidate candidates for specific markers and therapeutic targets. Thus, further studies for the characterisation of CSCs in human bone and soft tissue sarcomas might contribute to the elucidation of the mechanisms of tumourigenesis and to the establishment of novel therapeutic strategies.

## Figures and Tables

**Table 1:** Tumorigenesis of SP and MP cells in NOD/SCID mice

	**Cell number for injection**			
	**1 × 10^2^**	**1 × 10^3^**	**1 × 10^4^**	**1 × 10^5^**
*MFH2003*				
SP cells	0/5	1/5	2/5	1/2
MP cells	0/5	0/5	0/5	1/2

SP and MP cells were isolated separately and injected into the backs of the subcutaneous space of NOD/SCID mice. Tumour formation was observed for 12 weeks after injection.

**Table 2:** List of genes upregulated in SP cells of MFH2003

				**Expression ratio (SP/MP)**	
**Gene symbol**	**Gene name**	**Accession no.**	**Gene ontology**	**Cy5/Cy3**	**Cy3/Cy5**
ANKRD11	Ankyrin repeat domain 11	NM_013275	Electron transport	2.1	2.7
SLC2A4	Solute carrier family 2, member 4	NM_001042	Carbohydrate transport	2.2	2.5
KIAA1440	KIAA1440	AB037861	Unknown	2.2	3.3
SURF6	Surfeit 6	NM_006753	Ribosome biogenesis	2.3	2.8
VPF	Vascular permeability factor	M27281	Cell proliferation	3.0	3.5
C20orf14	Chromosome 20 open reading frame 14	NM_012469	RNA processing	2.1	3.6
PHLDA3	Pleckstrin homology-like domain, family A, member 3	NM_012396	Physiological processes	2.1	2.4
ZNF19	Zinc finger protein 19	NM_152907	Regulation of transcription	2.8	2.4
MCL1	Myeloid cell leukaemia sequence 1	NM_021960	Apoptosis	2.1	3.9
APOE	Apolipoprotein E	NM_000041	Lipid transport	2.1	3.8
NR4A2	Nuclear receptor subfamily 4, group A, member 2	NM_006186	Regulation of transcription	2.5	4.1
IRX3	Iroquois-related homeobox 3	BC023667	Regulation of transcription	3.3	2.9
GNB3	Guanine nucleotide-binding protein, *β-*polypeptide 3	NM_002075	G-protein coupled receptor protein signaling	2.1	5.0
NLRP12	NLR family, pyrin domain containing 12	NM_144687	Apoptosis	2.1	2.3
PTN	Pleiotrophin	NM_002825	Neurogenesis	2.0	2.6
ABCG2	ATP-binding cassette, sub-family G, member 2	NM_004827	Transport	2.2	2.9
APOL1	Apolipoprotein L	NM_145343	Lipid transport	2.2	2.6
MDFI	MyoD family inhibitor	NM_005586	Unknown	2.8	3.1
PRSS15	Protease, serine, 15	NM_004793	ATP-dependent proteolysis	2.1	2.5
MSX1	Msh homeo box homolog 1	NM_002448	Regulation of transcription	2.1	2.8
LDLR	Low density lipoprotein receptor	NM_000527	Cholesterol metabolism	2.1	3.1
LMNA	Lamin A/C	NM_170707	Cellular morphogenesis	2.1	2.9
MVK	Mevalonate kinase	BC016140	Cholesterol biosynthesis	2.2	2.1

Genes showing the ratio more than 2.0, which were reproducible in two experiments, were listed.
